# Crystal structure of the Rho-associated coiled-coil kinase 2 inhibitor belumosudil bound to CK2α

**DOI:** 10.1107/S2053230X22008767

**Published:** 2022-09-26

**Authors:** Paul Brear, Marko Hyvönen

**Affiliations:** a Department of Biochemistry, University of Cambridge, 80 Tennis Court Road, Cambridge, United Kingdom; Bristol-Myers Squibb, USA

**Keywords:** belumosudil, CK2α inhibition, Rho-associated coiled-coil kinase 2, ROCK2 inhibition, kinase inhibitors, selective inhibitors

## Abstract

The small molecule belumosudil was initially identified as a selective inhibitor of Rho-associated coiled-coil kinase 2 (ROCK2) and has recently been approved for the treatment of graft-versus-host disease. However, recent studies have shown that many of the phenotypes displayed upon treatment with belumosudil were due to CK2α inhibition. CK2α is in itself a very promising therapeutic target for a range of conditions and has recently been put forward as a potential treatment for COVID-19. Belumosudil presents a promising starting point for the development of future CK2α inhibitors as it provides a safe, potent and orally bioavailable scaffold. Therefore, several of the major hurdles in drug development have already been overcome. Here, the crystal structure of belumosudil bound to the ATP site of CK2α is presented. This crystal structure combined with modelling studies further elucidates how belumosudil could be developed into a selective and potent CK2α or ROCK2 inhibitor.

## Abstract 2

The crystal structure of the Rho-associated coiled-coil kinase 2 (ROCK2) inhibitor belumosudil bound to CK2α suggests ways in which specificity for either ROCK2 or CK2α can be increased.

## 1. Introduction

The inhibition of kinases has great therapeutic potential as almost every cellular process is regulated by them. Indeed, as of 2021, 70 small molecules targeting kinases had been approved (Cohen *et al.*, 2021 ▸; Wu, Nielsen *et al.*, 2015 ▸). The inhibition of kinases can be achieved by a number of strategies (Wu, Clausen *et al.*, 2015 ▸; Panicker *et al.*, 2019 ▸; Lu *et al.*, 2020 ▸; Bain *et al.*, 2003 ▸). However, the most common approach is to target the ATP site, as it is a deep druggable pocket against which high-affinity inhibitors can rapidly be developed. Unfortunately, the main technical challenge of targeting the ATP site is one of selectivity (Davis *et al.*, 2011 ▸; Bain *et al.*, 2007 ▸). In brief, the geometry and chemical properties of the ATP site in all kinases are constrained by the need to bind ATP. Therefore, inhibitors that bind in the ATP site of one kinase are likely to bind in the ATP sites of many other kinases. A recent example of this is the case of the inhibitor belumosudil, which was first identified as a specific inhibitor of Rho-associated coiled-coil kinase 2 (ROCK2), with a *K*_d_ of 54 n*M* (Blair, 2021 ▸). ROCK2 plays a role in the pro- and anti-inflammatory immune-cell response (Zanin-Zhorov *et al.*, 2016 ▸). Indeed, belumosudil has been recently approved for the treatment of graft-versus-host disease, in which the transplanted cells from a bone-marrow transplant attack the host cells, causing an immune response (Blair, 2021 ▸). However, it was recently observed that belumosudil displayed a different phenotype to other ROCK2 inhibitors, showing an anti-adipogenic effect (Tran & Chun, 2021 ▸). This observation was followed up by an extensive kinase screening panel, which showed that belumosudil strongly inhibits 3% of kinases (Tran & Chun, 2021 ▸). Of these, the most potently inhibited kinase was CK2α, with a *K*_d_ of 128 n*M*. Other potently inhibited kinases included MRCKB (1% of control), BIKE (3.9% of control) and DMPK2 (3.9% of control). Further investigations revealed that the biological activity of belumosudil was in part due to its inhibition of CK2α. CK2α is an interesting target for inhibition for a number of reasons. Firstly, although it phosphorylates over 300 substrates and is therefore involved in most processes within the cell, it is also overexpressed by a number of cancers due to its anti-apoptotic role (Borgo *et al.*, 2021 ▸). This means that cancer cells can be significantly more sensitive to CK2α inhibition than noncancerous cells. Secondly, it has recently been shown that CK2α inhibition prevents viral replication in SARS-CoV-2 and other infections (Bouhaddou *et al.*, 2020 ▸; Miranda *et al.*, 2021 ▸; Borgo *et al.*, 2021 ▸). This opens up the possibility of using CK2α inhibitors in the treatment of a number of viral infections. To gain further understanding to determinants of inhibitor specificity (or lack thereof) and to aid the development of belumosudil as a potent and selective CK2α or ROCK2 inhibitor, we have determined the crystal structure of belumosudil bound to CK2α.

## 2. Experimental

### 2.1. Crystallization and soaking

Crystals of CK2α were grown using the previously developed methodology optimized for drug discovery and ligand screening (Brear *et al.*, 2016 ▸). Briefly, the CK2α_KA mutant was crystallized at 5 mg ml^−1^ in 20 m*M* Tris pH 8.0, 350 m*M* NaCl, 1 m*M* DTT, 25 m*M* ATP with 112.5 m*M* MES pH 6.5, 35% glycerol ethoxylate, 180 m*M* ammonium acetate in a 1:1 ratio with a total volume of 2 µl. The CK2α_KA mutant contains the mutations K74A, K75A, K76A and R21S. These mutations do not affect binding in the ATP site or the conformation of the ATP site (Brear *et al.*, 2020 ▸; De Fusco *et al.*, 2017 ▸; Iegre *et al.*, 2018 ▸; Brear *et al.*, 2016 ▸). These crystals were then soaked overnight with 10 m*M* belumosudil (MedChemExpress) in mother liquor with 10% DMSO. The crystals were flash-cooled in liquid nitrogen and stored under cryogenic conditions until data collection.

### 2.2. Structure determination

Data were collected from belumosudil-soaked CK2α crystals on beamline I04 at Diamond Light Source. The data were processed with the automated data-processing pipeline using *DIALS* (Winter *et al.*, 2018 ▸). The structure was solved by molecular replacement with *Phaser* (Winn *et al.*, 2011 ▸) using a previous CK2α structure from which ligands in the ATP site had been removed (PDB entry 5cvh; Brear *et al.*, 2016 ▸). The structure was refined using *REFMAC*5 (Winn *et al.*, 2011 ▸) with manual editing in *Coot* (Emsley *et al.*, 2010 ▸). Ligand constraints were generated using *grade* (Smart *et al.*, 2011 ▸). Data-collection and refinement statistics are given in Table 1 ▸.

### 2.3. Modelling with *GOLD*

Modelling was performed using the *GOLD* software package from The Cambridge Crystallographic Data Centre (CCDC; Jones *et al.*, 1997 ▸) based on the structure of CK2α bound to belumosudil as reported here (PDB entry 7z39). H atoms were added to the structure and the ligands were extracted. The active site was defined as a 10 Å sphere around the position of belumosudil. Ligand files were generated using *grade* (Smart *et al.*, 2011 ▸) and converted to Mol2 files using *Open Babel* (O’Boyle *et al.*, 2011 ▸). The results were visualized with *PyMOL* (Schrödinger). Modelling for ROCK2 was performed using the same method based on the structure of ROCK2 bound to 1426382-07-1 (PDB entry 4wot; Boland *et al.*, 2015 ▸).

## 3. Results

**Figure 1:**
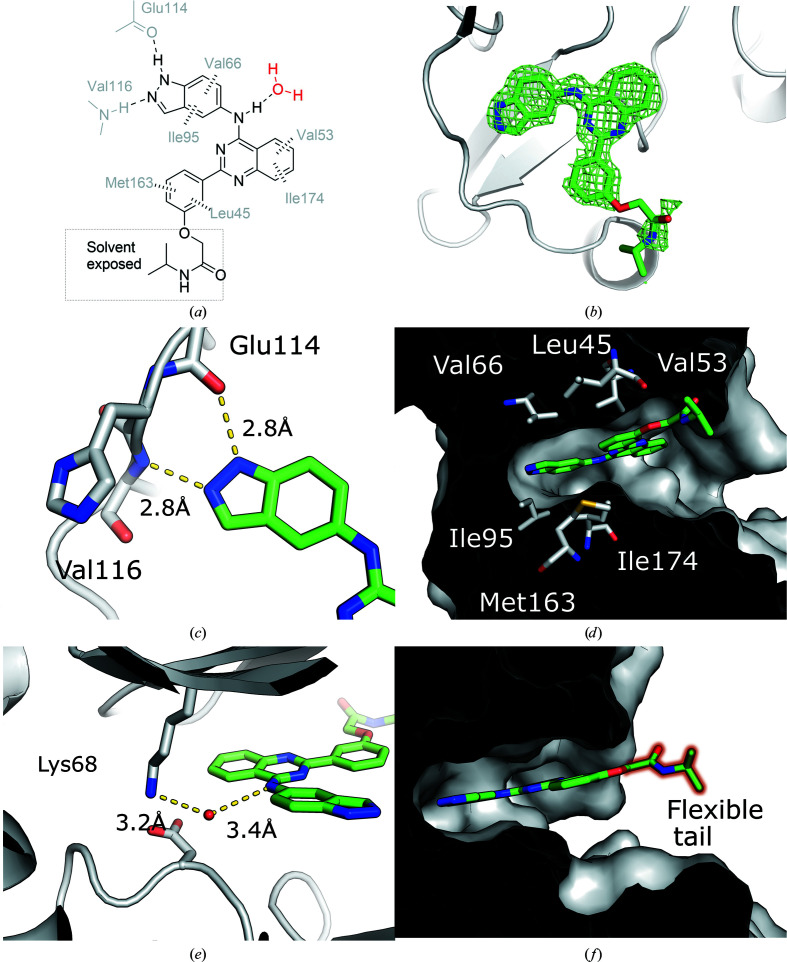
Belumosudil complexed with CK2α. (*a*) The interactions of belumosudil in the ATP site of CK2α observed in the crystal structure. Hydrogen bonds are shown as dashed lines and hydrophobic interactions with wider dashes. (*b*) The difference electron-density map in the CK2α ATP site, contoured at 2 r.m.s.d., before belumosudil was modelled. The refined structure of the inhibitor is superimposed for reference. (*c*) The interaction of belumosudil with the hinge region. (*d*) The hydrophobic residues of the ATP site that sandwich the aromatic ring systems of belumosudil. (*e*) The bridging water interaction between belumosudil and Lys68. (*f*) The linker to the flexible tail of belumosudil sticks out of the ATP site into solvent, with part of the tail unmodelled due to a lack of electron density.

The belumosudil-soaked crystal resulted in a co-crystal structure of the inhibitor bound to CK2α determined at a resolution of 1.60 Å (Fig. 1 ▸), with clear density for the inhibitor in the difference density map in the ATP site before belumosudil was modelled (Fig. 1 ▸ *b*). Belumosudil is composed of three ring systems and a flexible ‘tail’ on the phenyl group (ring 3; Fig. 1 ▸ *a*), with the difference map showing clear density for the majority of the ligand apart from the flexible tail. The indazole ring (ring 1) interacts with the hinge region of CK2α, with the N-1 (N4) and N-2 (N5) atoms of the indazole forming hydrogen bonds to the backbone carbonyl of Glu114 and the amide N atom of Val116, respectively (Fig. 1 ▸ *c*). The hydrophobic aromatic system of the indazole (ring 1) is sandwiched between Val66 and Ile95 (Fig. 1 ▸ *d*). The central quinazoline ring (ring 2) is sandwiched between Val53 and Ile174 (Fig. 1 ▸ *d*). The main contributor to the binding of many CK2α inhibitors is the interaction of Lys68 with a carboxylic acid in the inhibitor. In the belumosudil–CK2α complex a bridging water interaction between Lys68 and the amine N atom links the indazole and quinazoline moieties (Fig. 1 ▸ *e*). The phenyl group of belumosudil (ring 3) binds in the entrance of the ATP site, interacting with the hydrophobic residue Leu45 from the top and with Met163 underneath the ligand (Fig. 1 ▸ *d*). Finally, the amide-linked tail points out into solvent and does not appear to contribute to the binding (Fig. 1 ▸ *f*). Therefore, the electron density, although sufficient to define the likely position of the tail, is weakly defined with higher *B* factors.

## 4. Optimization of belumosudil for selectivity

This new co-crystal structure of belumosudil bound to CK2α suggests a number of strategies to increase its affinity for CK2α and improve its selectivity. Firstly, most high-affinity CK2α ligands interact directly with Lys68, and therefore recreating this interaction in belumosudil would be likely to increase the affinity for CK2α. However, Lys68 is conserved across the kinome, so targeting this residue may lead to selectivity issues. Secondly, this structure indicates that the flexible tail does not interact with the kinase and therefore could be removed or modified; for example, for the creation of a PROTAC-type inhibitor or attachment to a probe molecule.

**Figure 2:**
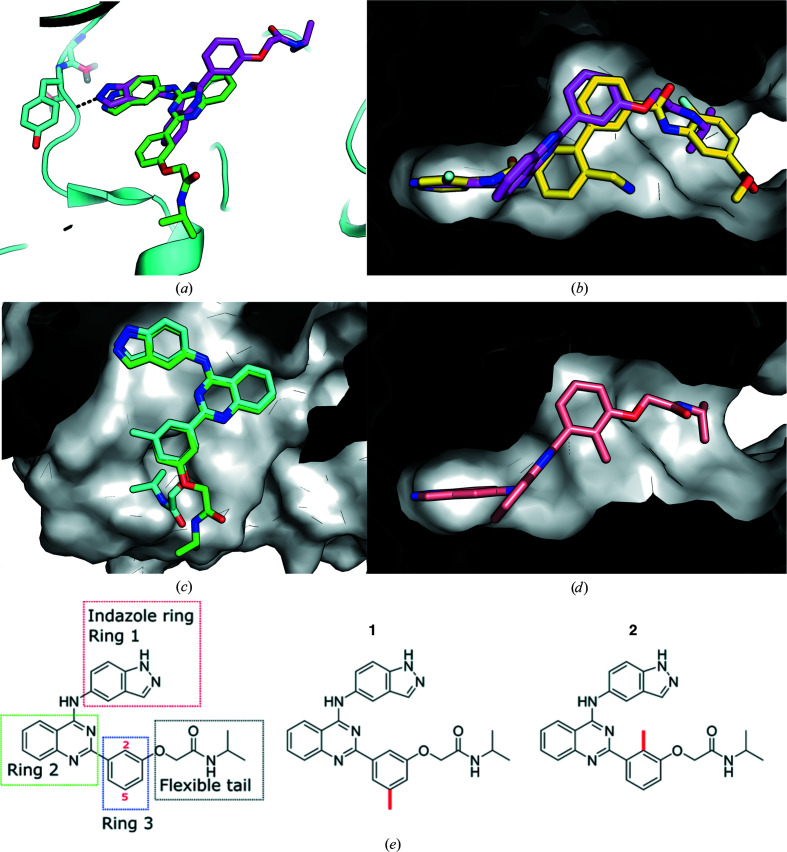
(*a*) A comparison of the predicted binding mode of belumosudil to ROCK2 (purple) and the binding mode of belumosudil to CK2α (green) observed in the crystal structure. (*b*) (i) The predicted binding mode of belumosudil (purple) to ROCK2 compared with the binding mode of 1426382-07-1 (yellow) to ROCK2 as observed in a crystal structure. (ii) Rotated by 180°. (*c*) The predicted binding mode of compound **1** to CK2α (cyan) compared with the binding mode observed in the crystal structure (green). (*d*) The predicted binding mode of compound **2** to ROCK2. (*e*) The structures of belumosudil and of the two analogues designed to have increased selectivity.

In the absence of a belumosudil-bound ROCK2 structure, the inhibitor was modelled into the ATP site of ROCK2 using the *GOLD* software package to allow the binding mode to be compared between ROCK2 and CK2α. As expected, the indazole ring (ring 1) of belumosudil is predicted to interact with the hinge region of ROCK2 as with CK2α (Fig. 2 ▸ *a*). Indeed, this hinge interaction is present in most known promiscuous kinase inhibitors. Comparison with the structures of selective kinase inhibitors (for example CAM4066; Brear *et al.*, 2016 ▸) reveals that the selective inhibitors generally have interactions outside of the conserved ATP site which are not present with belumosudil. Ring 2 is likely to bind in a similar position as in the CK2α complex, but it is flipped over and ring 3 is predicted to extend in the opposite direction compared with when bound to CK2α (Fig. 2 ▸ *a*). Although the predicted binding mode of belumosudil to ROCK2 is different to that seen in CK2α, it is similar to the binding mode of 1426382-07-1 (Fig. 2 ▸ *b*; PDB entry 4wot; Boland *et al.*, 2015 ▸) to ROCK2. The hinge-binding N atoms of both compounds are predicted to bind in the same position and the hydrophobic groups of both compounds are predicted to bind in the same pockets. Comparison of the predicted binding mode of belumosudil to ROCK2 with the structure of CK2α shows that this binding mode would be prevented in CK2α by steric clashes with Ser51 and Val53. These clashes force ring 3 to point into the opposite side of the ATP site (Fig. 2 ▸ *a*). In constrast, when belumosudil binds to ROCK2 ring 3 is predicted to stack between Leu221/Met172 and Leu155/Ala119. Likewise, the corresponding 3-chloro­pyridine group of 1426382-07-1 is also shown to bind in this pocket in the crystal structure. The similarities between the predicted binding modes of belumosudil and ROCK2 and the actual binding modes of 1426382-07-1 and ROCK2 indicate that the predicted binding mode of belumosudil to ROCK2 is likely to be accurate.

These models predict that the selectivity issues with belumosudil are likely to be related to the hinge region, as the binding mode is very conserved. However, the hinge inter­action tends to contribute significant energy to the binding, and therefore modifying the indazole ring (ring 1; Fig. 2 ▸ *e*) is not a promising strategy without a loss of potency. However, ring 3 is predicted to bind in a different position in the two proteins, in a small hydrophobic pocket in ROCK2 and at the mouth of the ATP site in CK2α, and this ring could be targeted to build in selectivity. We speculate that modification of position 5 in ring 3 would cause a steric clash with ROCK2, while modification of position 2 would disrupt the planarity of the ring 2/3 system that is required for CK2α binding. Modelling of methyl substitutions at these positions into the ATP site of CK2α and ROCK2 clearly shows how selectivity between these two proteins could be engineered. A methyl group at position 5 (compound **1**) is predicted to prevent binding to ROCK2, as the substituents would not be accommodated in the hydrophobic pocket, while still being compatible with CK2α binding (Fig. 2 ▸ *c*). On the other hand, a methyl group at position 2 of ring 2 (compound **2**) is predicted to disrupt the planar ring system that is needed for the binding to CK2α while locking the inhibitor in the predicted pose required for binding to ROCK2.

Belumosudil provides a valuable starting point for the development of selective CK2α inhibitors as it provides a scaffold that has already been proven to be safe and effective in humans. Furthermore, belumosudil has a good oral bio­availability of 65% and it is stable to metabolism with a half-life of 19 h (Blair, 2021 ▸). These two characteristics mean that belumosudil can be administered orally once a day, a greatly sought-after property in a drug molecule. This crystal structure of belumosudil bound to CK2α can guide the further development of belumosudil, and through our modelling studies we have suggested ways in which it could be optimized into a more selective CK2α or ROCK2 inhibitor. These small changes are unlikely to change the absorption, distribution, metabolism and excretion (ADME) characteristics sufficiently to affect the oral availability and other properties of the compound. This work also demonstrates the general value of determining structures of inhibitors with cross-reactive targets, providing insights into the mechanism by which promiscuity arises and suggesting ways in which it could be eliminated.

## Supplementary Material

Modelling results for CK2. DOI: 10.1107/S2053230X22008767/rf5038sup1.bin

Modelling results for ROCK2. DOI: 10.1107/S2053230X22008767/rf5038sup2.bin

PDB reference: belumosudil bound to CK2α, 7z39

## Figures and Tables

**Table 1:** Data-collection and refinement statistics for the structure of belumosudil bound to CK2α

PDB code	7z39
Beamline	I04, Diamond Light Source
Wavelength (Å)	0.979507
Data processing	
Resolution range (Å)	36.07–1.60 (1.63–1.60)
Space group	*P*12_1_1
*a*, *b*, *c* (Å)	58.51, 45.75, 63.46
α, β, γ (°)	90.0, 112.5, 90.0
Total reflections	459559 (15594)
Unique reflections	39677 (3446)
Multiplicity	11.6 (9.7)
Completeness (%)	96.28 (84.32)
Mean *I*/σ(*I*)	20.7 (1.7)
Wilson *B* factor (Å^2^)	20.79
*R*_merge_	0.064 (0.733)
*R*_meas_	0.070 (0.812)
*R*_p.i.m._	0.028 (0.349)
CC_1/2_	0.999 (0.815)
Refinement	
Resolution range (Å)	50.52–1.60 (1.641–1.600)
Reflections used in refinement	37696 (2320)
Reflections used for *R*_free_	1885 (134)
*R*_work_	0.1638 (0.246)
*R*_free_	0.2126 (0.266)
No. of non-H atoms	
Total	3153
Macromolecules	2819
Ligands	55
Solvent	279
Protein residues	327
R.m.s.d., bond lengths (Å)	0.014
R.m.s.d., angles (°)	1.85
Ramachandran favoured (%)	95.69
Ramachandran allowed (%)	4.31
Ramachandran outliers (%)	0
Rotamer outliers (%)	3.25
Clashscore	4.96
Average *B* factor (Å^2^)	
Overall	27
Macromolecules	26
Belumosudil	40
Other ligands	47
Solvent	34
